# Improving magnetic properties of Mn- and Zn-doped core–shell iron oxide nanoparticles by tuning their size

**DOI:** 10.3762/bjnano.16.157

**Published:** 2025-12-15

**Authors:** Dounia Louaguef, Ghouti Medjahdi, Sébastien Diliberto, Klaus M Seemann, Thomas Gries, Joelle Bizeau, Damien Mertz, Eric Gaffet, Halima Alem

**Affiliations:** 1 Université de Lorraine, CNRS, IJL, F54011 Nancy, France; 2 Institut de physique et de chimie des matériaux, UMR 7504 CNRS, Université de Strasbourg, France

**Keywords:** core–shell nanoparticles, magnetic hyperthermia, magnetic properties

## Abstract

Superparamagnetic iron oxide nanoparticles (SPIONs) offer promising applications in nanomedicine due to their appealing properties. Their magnetic and magnetic hyperthermia properties are considered as relevant tools for low invasive cancer therapeutic applications. In this work, we report on the synthesis of polyhedral core–shell SPIONs. Their size was tuned to improve their magnetic properties. Furthermore, by hybridizing into a core–shell inorganic/inorganic structure, the nanoparticles can achieve significantly improved magnetic-to-thermal energy conversion efficiency (at least tenfold). The designed core NPs are composed of a Zn_0.4_Fe_2.6_O_4_ core and a MnFe_2_O_4_ shell. Their size and morphology were determined by transmission electron microscopy, Fourier-transform infrared spectroscopy was used to investigate their chemical composition. The iron oxide phase was confirmed by Mössbauer analysis, and the magnetic properties were studied to select the ideal size for magnetic hyperthermia application.

## Introduction

Magnetic nanoparticles have emerged as a versatile class of materials due to their unique magnetic properties, small size, and biocompatibility, which enable them to be used in a wide range of biomedical applications. These applications include magnetic resonance imaging, magnetic separation, targeted drug delivery, and hyperthermia [1–2]. Magnetic hyperthermia has been extensively investigated as a novel cancer treatment due to its ability to locally generate heat in tumors, thereby minimizing damage to healthy tissues compared to conventional chemotherapy and radiotherapy [3]. This process relies on exposing magnetic nanoparticles to an alternating magnetic field, causing them to generate heat through Brownian and Néel relaxation mechanisms [4]. The heat generation capacity of these nanoparticles is often quantified by the specific absorption rate (SAR), which represents the power dissipated as heat per gram of nanoparticles under the influence of an alternating magnetic field.

The development of magnetic nanoparticles with enhanced SAR and improved biocompatibility has been a major objective in the field of nanomedicine. Among the various materials used for magnetic hyperthermia, superparamagnetic iron oxide nanoparticles (SPIONs), such as magnetite (Fe_3−δ_O_4_) and maghemite (γ-Fe_2_O_3_), have been extensively studied due to their low toxicity, biocompatibility, and ease of synthesis [5]. However, SPIONs face several challenges related to their relatively low saturation magnetization (*M*_s_) and specific loss power (SLP), which limit their effectiveness in hyperthermia applications. Consequently, efforts have been made to design new ferrite systems that offer improved performance. One promising strategy involves substituting iron with other divalent metal cations (M) in spinel ferrites (MFe_2_O_4_), such as cobalt (Co), manganese (Mn), zinc (Zn), and nickel (Ni), to modify the magnetic properties of the nanoparticles [6].

A significant amount of research has focused on CoFe_2_O_4_ as a shell material to enhance the magnetic properties of core–shell nanoparticles due to its high coercivity and magnetic anisotropy [7]. The formation of CoFe_2_O_4_ shells on Fe_3−δ_O_4_ cores has been shown to increase the magnetic saturation and improve hyperthermia performance. However, the cytotoxicity of Co-based nanoparticles poses a significant challenge for biomedical applications. Studies have demonstrated that Co^2+^ ions released from these particles can enter the bloodstream and accumulate in organs, where they induce oxidative stress, genotoxic effects, and inflammation [8–9]. This has raised concerns regarding their use in clinical applications, leading researchers to develop alternative, non-toxic substitutes for Co-based nanoparticles.

In this context, manganese ferrite (MnFe_2_O_4_) has been identified as a promising alternative due to its biocompatibility, high magnetic moment, and potential for use in hyperthermia and drug delivery applications [10]. Manganese is naturally present in the human body and exists primarily as Mn^2+^ and Mn^3+^ ions, making it more biocompatible than cobalt. The substitution of Fe^3+^ with Mn^2+^ in ferrites introduces uncompensated magnetic moments, thereby enhancing the overall magnetic properties [11]. MnFe_2_O_4_ nanoparticles have been reported to exhibit higher SAR and SLP values than Fe_3−δ_O_4_ or ZnFe_2_O_4_ due to the higher magnetic moment of Mn^2+^ ions [10].

While the chemical composition of magnetic nanoparticles is critical, the morphology of the particles also plays a significant role in determining their magnetic properties. Although spherical nanoparticles have been extensively studied, recent research suggests that polyhedral nanoparticles exhibit superior magnetic performance due to their reduced surface spin disorder [7]. Polyhedral NPs exhibit higher magnetic saturation, less spin canting, and higher SAR values than spherical NPs. For instance, Kasparis et al. demonstrated that polyhedral Zn_0.4_Fe_2.6_O_4_ NPs exhibited SAR values more than double those of their spherical counterparts [12]. This observation highlights the importance of morphology control in the design of high-performance magnetic nanoparticles.

One effective approach to achieving both enhanced magnetic properties and biocompatibility is the formation of core–shell structures, where the core provides a strong magnetic moment and the shell enhances magnetic anisotropy and stability. Core–shell NPs offer a pathway to improving SLP, SAR, and stability under physiological conditions [13]. The core–shell configuration allows for the tuning of magnetic properties through the choice of core and shell materials. While CoFe_2_O_4_ shells are known to increase magnetic anisotropy, their cytotoxicity limits their use. In contrast, MnFe_2_O_4_ shells provide a non-toxic alternative that enhances the overall magnetic performance while maintaining biocompatibility.

The orginality of this study lies in the synthesis and characterization of polyhedral Zn_0.4_Fe_2.6_O_4_ nanoparticles and their subsequent transformation into Zn_0.4_Fe_2.6_O_4_@MnFe_2_O_4_ core–shell nanoparticles with tunable sizes. Previous studies have explored core–shell systems with Co-based shells, but our approach employs MnFe_2_O_4_ as the shell material to eliminate the cytotoxicity concerns associated with cobalt. Furthermore, we demonstrate size control through synthesis by varying the concentration of oleic acid, a surfactant that influences particle size and morphology. This approach enables the synthesis of NPs with sizes ranging from 5 to 50 nm, with clear evidence of core–shell formation.

In summary, this study presents a novel approach to the synthesis of core–shell Zn_0.4_Fe_2.6_O_4_@MnFe_2_O_4_ nanoparticles with controlled size and morphology. By incorporating a MnFe_2_O_4_ shell, we achieve significant improvements in magnetic performance while expecting good biocompatibility. The use of size-controlled synthesis enables the exploration of size-dependent magnetic properties, while the direct characterization of the core–shell structure using Fourier-transform infrared spectroscopy (FTIR) and high-resolution transmission electron microscopy (HRTEM) provides a comprehensive understanding of the material’s composition and properties. This work paves the way for the development of next-generation biocompatible magnetic nanoparticles for cancer therapy and other biomedical applications.

## Materials and Methods

### Materials

Iron acetylacetonate, manganese acetylacetonate and oleic acid were purchased from Aldrich. Zinc acetylacetonate was purchased from Merck KGaA. Benzyl ether 99% from Acros organics. And finally, ethanol absolute anhydrous and toluene were purchased from Carlo ERBA. All the reactants were used as received.

### Synthesis

#### Synthesis of polyhedral nanoparticles

The core of nanoparticles (Zn_0.4_Fe_2.6_O_4_) was obtained by mixing 194.25 mg of iron(III) acetylacetonate (0.55 mmol) and 221.11 mg of Zn(II) acetylacetonate (0.83 mmol) in the presence of oleic acid (the amount of oleic acid was tune depending on the targeted size of the NPs (see Table 1) and 52.61 mL of benzyl ether (276.77 mol) [14–15]. The mixture was heated under an argon atmosphere (flow rate of ≈100 mL·min^−1^) at a rate of 5 °C·min^−1^ to 290 °C and maintained at this temperature for 30 min. After cooling to room temperature under ambient conditions, ethanol (100 mL) was added to precipitate the nanoparticles. The resulting black solid was collected by centrifugation (10,000 rpm, 10 min), washed twice with ethanol, and redispersed in toluene (10 mL) [7].

**Table 1:** Volume of oleic acid as a function of NPs targeted average diameter.

Core NPs average diameter (nm)	Core–shell NPs average diameter (nm)	Oleic acid volume (mL)

5	10	6.3
10	15	5.6
18	22	4.2
45	50	0.7

#### Synthesis of polyhedral core–shell nanoparticles

The polyhedral Zn_0.4_Fe_2.6_O_4_ nanoparticles were coated with an MnFe_2_O_4_ shell, targeted to be 5 nm in thickness, by mixing iron(III) acetylacetonate (0.18 mmol) and manganese(II) acetylacetonate (0.33 mmol) in the presence of oleic acid (see Table 1) and benzyl ether (52.5 mmol). After adding the Zn_0.4_Fe_2.6_O_4_ nanoparticles suspended in hexane, the mixture was heated in an argon atmosphere (flow rate of ca. 100 mL·min^−1^) at a rate of 5 °C·min^−1^ to 290 °C and maintained at this temperature for 30 min. After cooling to room temperature under ambient conditions, ethanol (100 mL) was added to precipitate the nanoparticles. The resulting black solid was collected by centrifugation (10,000 rpm, 10 min), washed twice with ethanol, and redispersed in toluene (10 mL) [7].

### Characterization methods

Structural characterization of the NPs was performed by X-ray diffractometry (XRD) measurements. X-ray diffraction patterns of NPs (Figure S1, Supporting Information File 1) were obtained with a PANalytical X’Pert Pro MPD diffractometer. The latter was used in a Bragg–Brentano configuration in reflection equipped with a Cu anticathode (Cu Kα radiation, λ = 0.154 nm) and a high-speed multichannel X’Celerator detector. The sample were placed on a silicon zero-background sample holder. The latter was installed on a rotating spinner to allow the highest number of grains to be in diffraction position, and the XRD patterns were recorded at room temperature. The nanoparticles were annealed at 300 °C to remove all the traces of the solvent. in an Anton Paar 1200N oven.

Mössbauer analysis (Figure S2, Supporting Information File 1) was performed to determine the NPs phase by using a conventional Mössbauer spectrometer in a standard transmission configuration at room temperature. The velocity scale was calibrated with a CoRh (25 mCi) source and a metallic iron foil. The evaluation of the Mössbauer spectra was performed by least-square fitting of lines using the Winnormos (Wissel) program. The error on all Fe Mössbauer spectra was ±0.1 mm·s^−1^.

High-resolution transmission electron microscopy (HRTEM) and scanning transmission electron microscopy (STEM) images were performed on a JEOL JEM-ARM 200F cold-FEG microscope operating at 200 kV and equipped with a spherical aberration probe corrector (Cs). The chemical compositions were determined by energy-dispersive X-ray spectroscopy The elemental maps were recorded on a SDD, Jeol DRY SD 30 GV X-ray spectrometer.

NP shapes and sizes were obtained using a CM200 TEM from Philips with an acceleration voltage of 200 kV, a point resolution of approximately 0.27 nm and a magnification from 50.000× to 750.000×. One drop of a diluted solution of NPs in toluene was deposited on a holey carbon grid. The latter was heated on a hot plate at 50 °C for 2 h to remove all solvent traces.

The chemical compositions of both core and core–shell NPs were investigated by Fourier-transform infrared spectroscopy (FTIR). Both spectra were recorded using a commercial Agilent FTIR 680 spectrometer in attenuated total reflection (ATR) mode. The spectra were acquired from 400 to 4000 cm^−1^ with a spectral resolution of 4 cm^−1^. Reference spectra were acquired before each measurement to determine the absorption spectra under ambient conditions. Each measurement was averaged over 200 scans in continuous mode to improve the signal-to-noise ratio.

Magnetic properties of the NPs were studied using a vibrating sample magnetometer (VSM) from Microsense applying a magnetic field of 5 kOe. Several milligrams of purified and surfactant-free NPs were encapsulated into a quartz-glass cup and hermetically sealed.

SAR measurements were performed using a calorimetric method on a DM 100 instrument and DM applicator (Nanoscale Biomagnetics™, Zaragoza, Spain) using MaNIaC software (Nanoscale Biomagnetics™). 1 mL of a solution containing the NPs dispersed in toluene (5 mg/mL) was placed in vials adapted for such measurements, and an alternating magnetic field of 536.5 kHz/300 G or 796 kHz/200 G was applied. The increase of temperature was recorded over a period of 60 s. The SAR values were then calculated following the method described by Perigo and colleagues [16]. The curve Δ*T* = *f*(*t*) was fitted with a second-order polynomial function of the form *y* = *a* + *b*_1_·*x* + *b*_2_·*x*^2^, where *b*_1_ corresponds to [d*T*/d*t*]*_t_*_=0_. The SAR value was finally calculated with the following equation:


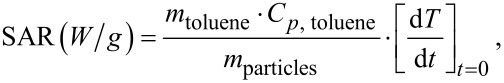


where *m*_toluene_ and *C_p_*_, toluene_ represent the mass (g) and the heat capacity (J·g^−1^·K^−1^) of the sample. *m*_particles_ (g) denotes the mass of the magnetic nanoparticles present in the sample and [d*T*/d*t*]*_t_*_=0_ is the derivative function of the temperature at *t* = 0 (K·s^−1^).

## Results and Discussion

### X-ray diffraction analysis

The XRD pattern (Supporting Information File 1, Figure S1) displays sharp and intense peaks characteristic of a well-crystallized material. The most intense peak is located at 2θ ≈ 35.5°, which corresponds to the (311) reflection of spinel ferrites. Additional peaks were observed at 2θ ≈ 18.3°, 30.1°, 43.2°, 53.5°, 57.1°, and 62.7°, corresponding to the (111), (220), (400), (422), (511), and (440) planes, respectively. These reflections are in good agreement with the standard spinel cubic phase (space group 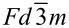), matching well with reference data such as JCPDS card no. 22-1012 for ZnFe_2_O_4_. No impurity phases were detected, indicating that the synthesized Zn_0.4_Fe_2.6_O_4_ is a single-phase spinel ferrite. The shift and relative intensity of the peaks may reflect the partial substitution of Fe^3+^ ions by Zn^2+^ ions, which influence the lattice parameters without disrupting the spinel framework.

### HRTEM observations

**Figure 1:**
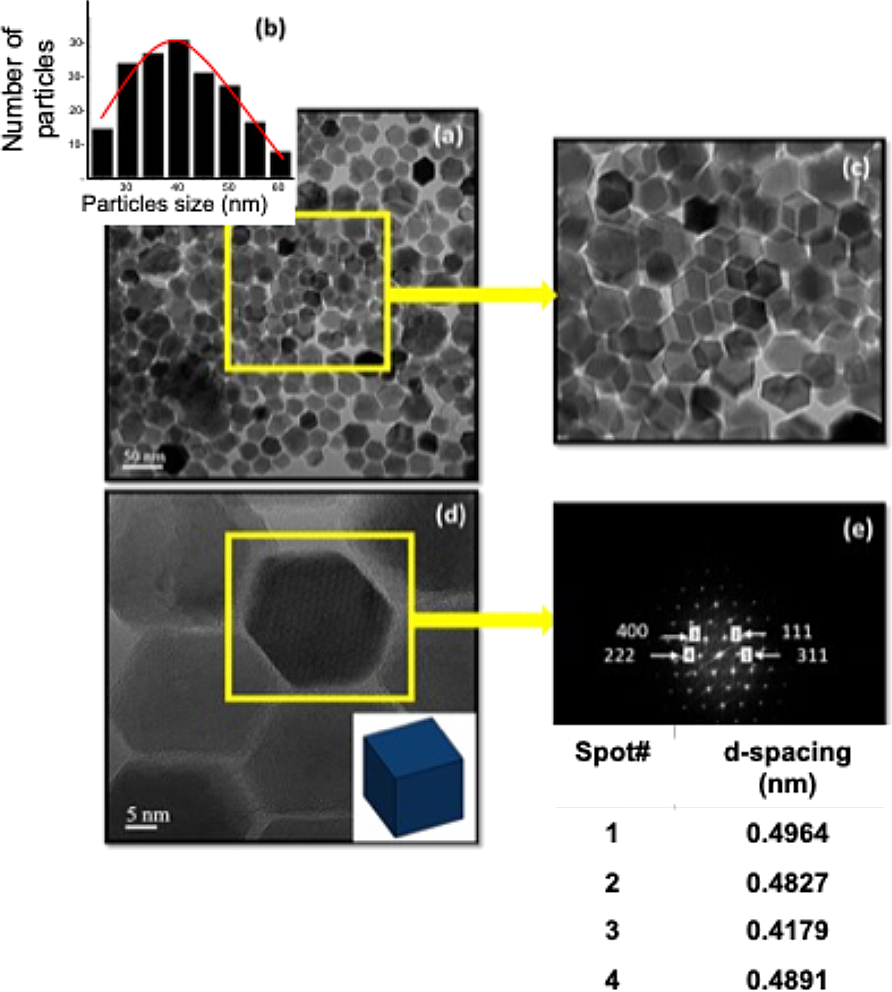
(a) TEM images (bright field) of Zn_0.4_Fe_2.6_O_4_ NPs (40 nm). (b) Size distribution histogram of Zn_0.4_Fe_2.6_O_4_ NPs. (c) Zoom on the framed part in yellow (a). (d) HRTEM image of Zn_0.4_Fe_2.6_O_4_ NPs. (e) Diffraction pattern of the framed part in yellow (d).

The primary objective of this study was to synthesize core–shell nanoparticles (NPs) with polyhedral morphology and enhanced magnetic properties. The synthesis approach focused on precise control of shape and size, critical factors for tuning magnetic behavior. By employing thermal decomposition, Zn_0.4_Fe_2.6_O_4_ NPs were synthesized with a well-defined polyhedral morphology, as confirmed by high-resolution transmission electron microscopy (HRTEM) (Figure 1). This morphology is critical as polyhedral-shaped NPs have been shown to exhibit superior magnetic properties compared to spherical particles due to increased surface area and facet effects [17].

High-resolution TEM images (Figure 1a) reveal well-defined, monodisperse polyhedral Zn_0.4_Fe_2.6_O_4_ nanoparticles with an average size of approximately 40 nm. The size distribution histogram (Figure 1b) confirms the uniformity of particle sizes, while a magnified image (Figure 1c) highlights the polyhedral morphology. The selected-area electron diffraction pattern (Figure 1d) exhibits distinct diffraction spots that can be indexed to the (222), (400), (111), and (311) planes of a spinel-type structure, in agreement with the X-ray diffraction (XRD) data (Figure S1, Supporting Information File 1). The presence of the (222) reflection, which is forbidden in XRD for a perfect spinel (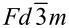) due to structure factor constraints, can be attributed to dynamic scattering effects or local structural ordering, as frequently observed in electron diffraction studies of ferrites. These features collectively indicate a high degree of crystallinity, consistent with previous reports on spinel ferrite nanoparticles [18].

**Figure 2:**
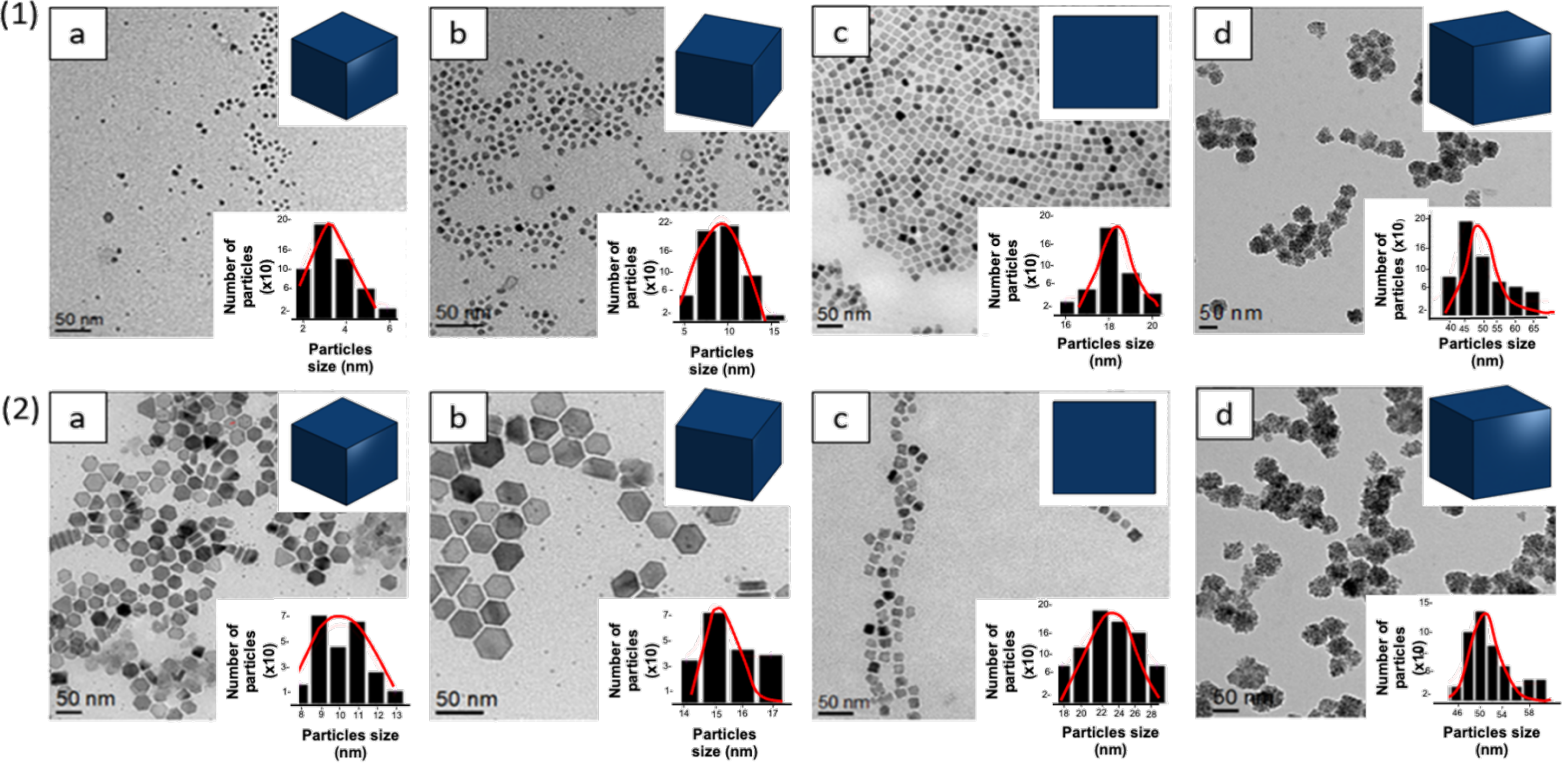
(1) TEM images of Zn_0.4_Fe_2.6_O_4_ NPs (bright-field images): 5 nm (a), 10 nm (b), 18 nm (c) and 45 nm (d). (2) TEM images of Zn_0.4_Fe_2.6_O_4_@MnFe_2_O_4_ NPs: 10 nm (a), 15 nm (b), 22 nm (c) and 50 nm (d).

To enhance the magnetic properties of Zn_0.4_Fe_2.6_O_4_ and Zn_0.4_Fe_2.6_O_4_@MnFe_2_O_4_ NPs based on their size, four distinct sizes were selected as shown in Table 2. These sizes were achieved by adjusting the ratio of oleic acid used during the synthesis of Zn_0.4_Fe_2.6_O_4_ and Zn_0.4_Fe_2.6_O_4_@MnFe_2_O_4_ (see Table 1). The target average size of these NPs and their polyhedral morphology were confirmed through transmission electron microscopy images (Figure 2). The labeled diffraction spots correspond to specific crystallographic planes (e.g., (400), (222), (111), and (311)), confirming the crystalline nature of the nanoparticles. The table (inset in the figure) lists the *d*-spacing values (in nm), which are consistent with the spinel structure of ZnFe_2_O_4_.

**Table 2:** Statistical analysis of Zn_0.4_Fe_2.6_O_4_ and Zn_0.4_Fe_2.6_O_4_@MnFe_2_O_4_ NPs diameters measured from TEM images (the number of measured particles varies from *n* = 260 to *n* = 640). Mean diameter, standard deviation (SD) and relative standard deviation (RSD) are shown.

Zn_0.4_Fe_2.6_O_4_				Zn_0.4_Fe_2.6_O_4_@MnFe_2_O_4_			
Targetted diameter (nm)	Mean diameter (nm)	Standard deviation (nm)	RSD^a^ (%)	Targetted diameter (nm)	Mean diameter (nm)	Standard deviation (nm)	RSD^a^ (%)

5 (Figure 2.1a)	4.5	1.9	42.49	10 (Figure 2.2a)	9.5	2	21.35
10 (Figure 2.1b)	8.9	1.6	18.98	15 (Figure 2.2b)	15.7	1.1	7.27
18 (Figure 2.1c)	18.1	1	5.72	22 (Figure 2.2c)	23	2.5	10.97
45 (Figure 2.1d)	50	7.5	15	50 (Figure 2.2d)	50.7	2.2	4.36

^a^Relative standard deviation.

**Figure 3:**
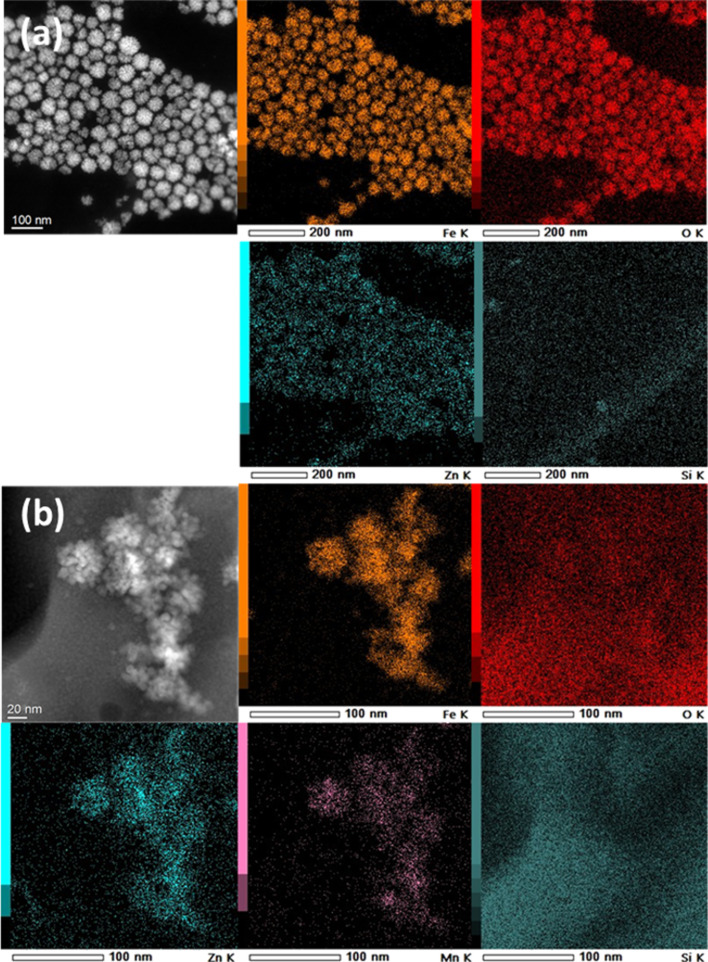
X-ray element mapping images of Zn_0.4_Fe_2.6_O_4_ NPs (45 nm) (a) and Zn_0.4_Fe_2.6_O_4_@MnFe_2_O_4_ NPs (50 nm) (b).

Elemental analysis using HRTEM with energy-filtered transmission electron microscopy (EELS) confirmed the elemental distribution within the core and shell of the NPs. As shown in Figure 3, Fe, Zn, and O are homogeneously distributed in the Zn_0.4_Fe_2.6_O_4_ NPs (Figure 3a). For Zn_0_._4_Fe_2.6_O_4_@MnFe_2_O_4_ NPs (Figure 3b), a clear distinction is observed, where Mn is exclusively located in the shell. This confirms the successful deposition of the MnFe_2_O_4_ shell on the Zn_0.4_Fe_2.6_O_4_ core. Such shell formation has been reported in other studies on core–shell NPs, where the selective deposition of the shell material has been achieved through precise control of synthesis conditions [19].

### FTIR analysis

**Figure 4:**
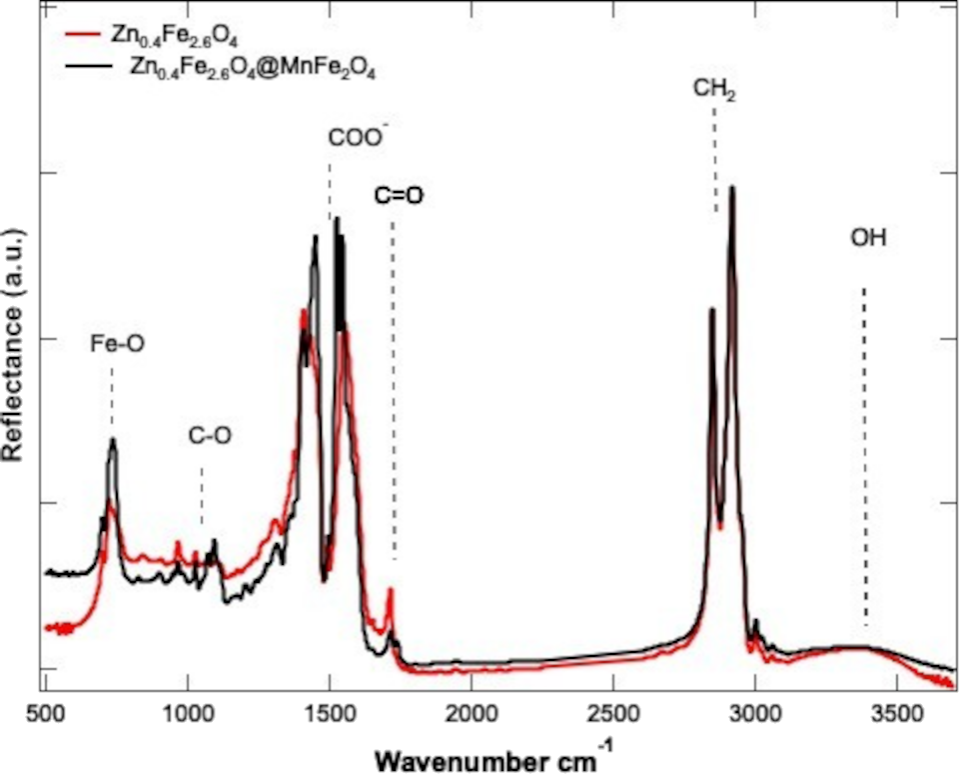
FTIR spectra of Zn_0.4_Fe_2.6_O_4_ NPs (45 nm in red) and Zn_0.4_Fe_2.6_O_4_@MnFe_2_O_4_ (50 nm in black).

The FTIR analysis of Zn_0.4_Fe_2.6_O_4_@MnFe_2_O_4_ and Zn_0.4_Fe_2.6_O_4_ NPs provides valuable insights into their structural properties and confirms the successful formation of both core and core–shell structures (Figure 4). The Fe–O stretching vibrations in spinel ferrites typically appear within the range of 500–650 cm^−1^, with tetrahedral Fe–O bonds generally observed around 550–600 cm^−1^. However, in the present study, the Zn_0.4_Fe_2.6_O_4_ sample exhibits a peak at approximately 715 cm^−1^, significantly higher than expected, and this peak shifts further to 730 cm^−1^ in the Zn_0.4_Fe_2.6_O_4_@MnFe_2_O core–shell composite. The higher vibrational frequency in Zn_0.4_Fe_2.6_O_4_ can be attributed to the partial substitution of Zn^2+^ into tetrahedral sites, which strengthens the Fe^3+^–O bond through enhanced covalency and changes in cation distribution, leading to shorter and stiffer bonds. The additional upward shift to 730 cm^−1^ in the core–shell structure reflects interfacial strain and electronic interactions between the Zn_0.4_Fe_2.6_O_4_ core and the MnFe_2_O shell. This strain distorts the tetrahedral FeO_4_ units, further stiffening the Fe–O bonds, while electronic coupling and potential cation redistribution at the interface increase the vibrational frequency. These structural and electronic effects collectively explain the observed peak positions and their shift, highlighting the impact of lattice distortions, bond strengthening, and interfacial interactions on the vibrational properties of the material [18]. The presence of peaks at ≈2850 cm^−1^ and ≈2920 cm^−1^ is associated with the C–H stretching vibrations from surface-capping agents, such as oleic acid, which is commonly used during synthesis to control particle size and prevent aggregation. The broad peak observed at ≈3400 cm^−1^ is linked to the O–H stretching vibrations of adsorbed water or hydroxy groups on the particle surface, which is a common feature for nanoparticles exposed to ambient conditions.

Although Zn and Mn are present in the Zn_0.4_Fe_2.6_O_4_ and Zn_0.4_Fe_2.6_O_4_@MnFe_2_O_4_ structures, the FTIR spectrum does not show distinct peaks associated with Zn–O or Mn–O bonds. This absence is due to the nature of the vibrational modes of Zn–O and Mn–O bonds. In spinel ferrites, the strong absorption bands corresponding to the Fe–O bonds at tetrahedral and octahedral sites are significantly more intense than the Zn–O and Mn–O vibrational modes, which have lower dipole moment changes during vibration. As a result, the Fe–O vibrations dominate the FTIR spectrum. Additionally, Zn^2+^ typically occupies tetrahedral sites in spinel structures, and its associated vibrational modes are often masked by the stronger Fe–O vibrations in this region. Mn^2+^, which is present in the shell of Zn_0.4_Fe_2.6_O_4_@MnFe_2_O_4_, also occupies octahedral sites and contributes to the overall bonding environment, but its distinct vibrational modes are often indistinguishable due to overlap with the Fe–O modes, especially since Fe^3+^ has a higher mass and stronger dipole moment change than Zn^2+^ and Mn^2+^.

### Magnetic properties of the core–shell NPs

**Figure 5:**
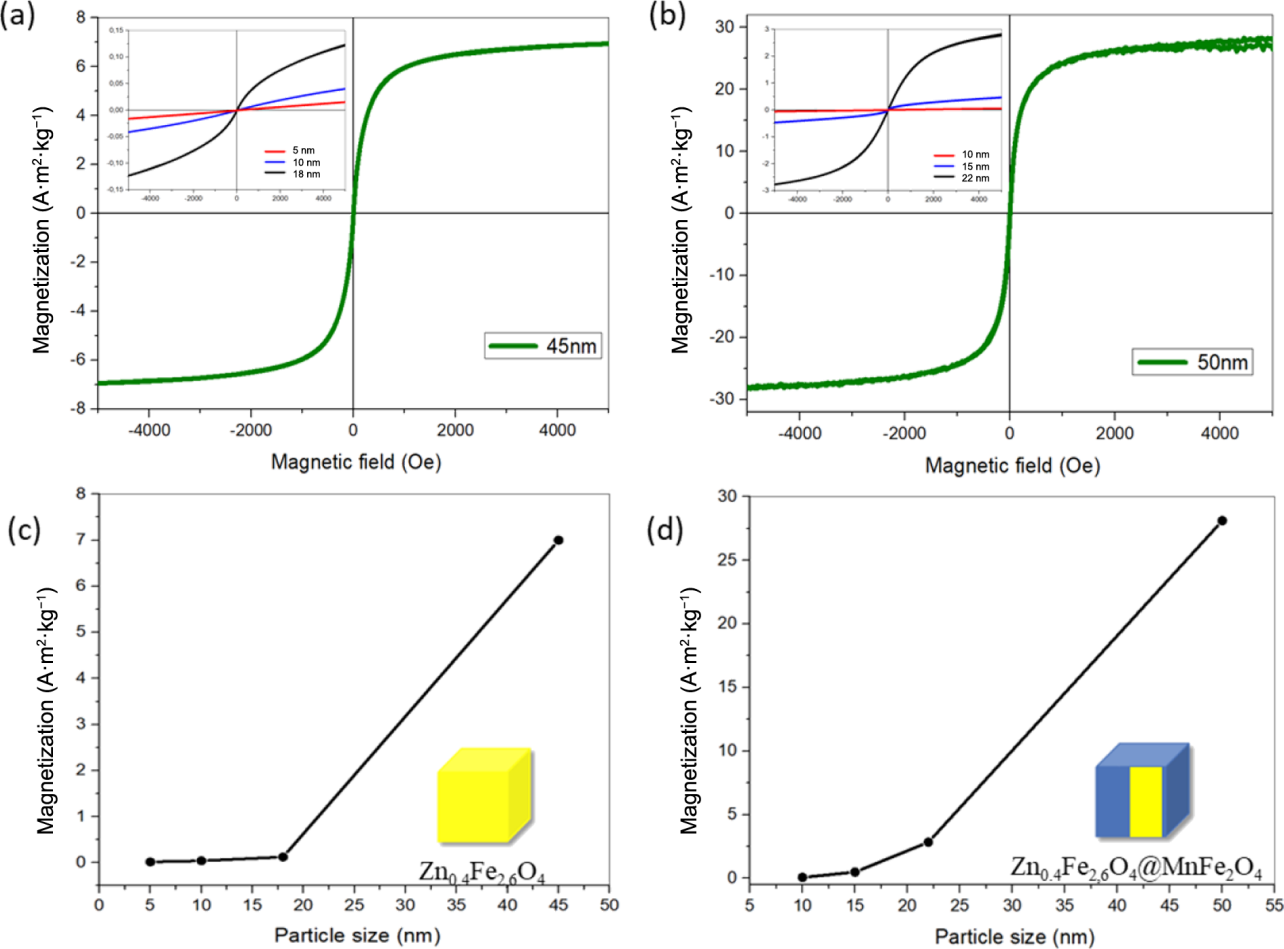
(a) Magnetization curves of Zn_0.4_Fe_2.6_O_4_ NPs. (b) Magnetization curves of Zn_0.4_Fe_2.6_O_4_@MnFe_2_O_4_ NPs. Maximal magnetization as a function of Zn_0.4_Fe_2.6_O_4_ NPs size (c). Maximal magnetization as a function of Zn_0.4_Fe_2.6_O_4_@MnFe_2_O_4_ NPs size (d). The magnetization values presented are normalized to the total sample mass.

The magnetic saturation as a function of magnetic field of Zn_0.4_Fe_2.6_O_4_ and Zn_0.4_Fe_2.6_O_4_@MnFe_2_O_4_ NPs are presented in Figure 5. The maximum applied field in this study was 5000 Oe. The *M*_s_ values of Zn_0.4_Fe_2.6_O_4_ NPs exhibit a clear size-dependent behavior, with larger particles showing significantly higher *M*_s_ values. The NPs with sizes of 5.0 ± 0.4, 10.0 ± 0.7, 18.0 ± 1.7, and 45.0 ± 1.2 nm exhibited *M*_s_ values of 0.012, 0.031, 0.12, and 7 A·m^2^·kg^−1^, respectively (Table 3). Similar trends have been reported in the literature, where larger particle sizes lead to higher *M*_s_ values due to the reduction of surface spin canting and the improved alignment of magnetic moments [18–20]. For instance, Wang et al. observed that ZnFe_2_O_4_ NPs with sizes ranging from 5 nm to 50 nm displayed *M*_s_ values increasing from 1.5 to 30 A·m^2^·kg^−1^, highlighting the critical role of particle size in magnetic properties [21].

**Table 3:** Magnetic saturation values of Zn_0.4_Fe_2.6_O_4_ and Zn_0.4_Fe_2.6_O_4_@MnFe_2_O_4_ NPs. The magnetization values presented are normalized to the total sample mass.

Zn_0.4_Fe_2.6_O_4_	Magnetic saturation [A·m^2^·kg^−1^]	Zn_0.4_Fe_2.6_O_4_@MnFe_2_O_4_	Magnetic saturation [A·m^2^·kg^−1^]

5.0 ± 0.4 nm (red)	0.012	10.0 ± 1.1 nm (red)	0.06
10.0 ± 0.7 nm (blue)	0.031	15.0 ± 1.03 nm (blue)	0.49
18.0 ± 1.7 nm (black)	0.12	22.0 ± 1.02 nm (black)	2.84
45.0 ± 1.2 nm (green)	7.000	50.0 ± 1.0 nm (green)	28.12

The role of the core–shell structure in enhancing the magnetic properties of NPs is clearly evident in the case of 45.0 nm Zn_0.4_Fe_2.6_O_4_ NPs and 50.0 nm Zn_0.4_Fe_2.6_O_4_@MnFe_2_O_4_ NPs. In this study, the addition of an MnFe_2_O_4_ shell to the Zn_0.4_Fe_2.6_O_4_ core resulted in a more than fourfold increase in *M*_s_, from 7 A·m^2^·kg^−1^ for the core alone to 28.12 A·m^2^·kg^−1^ for the core–shell system.

Furthermore, the superparamagnetic behavior of both Zn_0.4_Fe_2.6_O_4_ and Zn_0.4_Fe_2.6_O_4_@MnFe_2_O_4_ NPs was confirmed by the absence of hysteresis in the magnetization curves. This behavior is characteristic of superparamagnetic NPs, where thermal energy is sufficient to overcome magnetic anisotropy energy barriers, leading to zero remanent magnetization when the applied field is removed. Superparamagnetism is a desirable property for biomedical applications, as it prevents particle aggregation in the absence of an external magnetic field.

### Magnetic hyperthermia analysis

**Figure 6:**
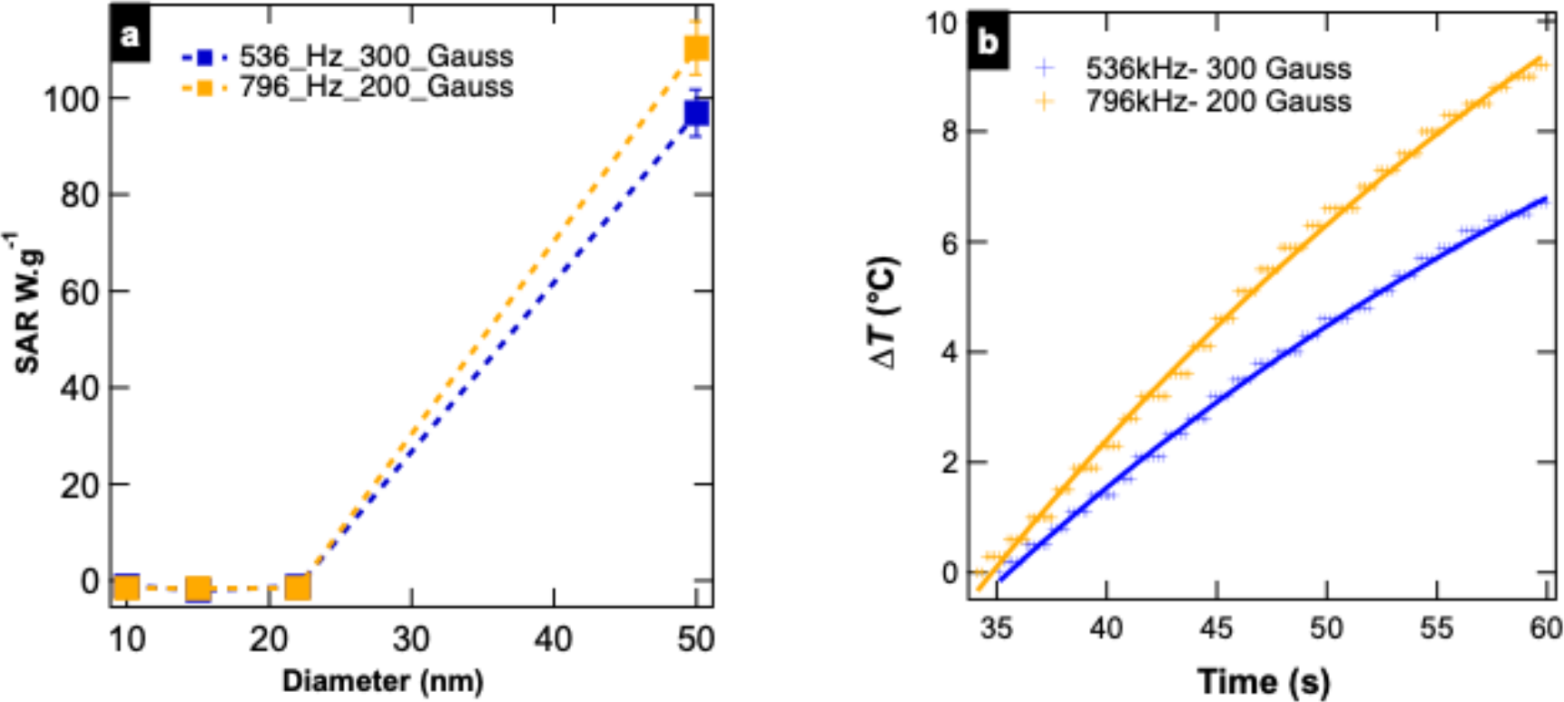
(a) SAR values of Zn_0.4_Fe_2.6_O_4_@MnFe_2_O_4_ NPs according to their different sizes. (b) Heating curves of 50 nm Zn_0.4_Fe_2.6_O_4_@MnFe_2_O_4_ as a function of time under an alternating magnetic field of 536.5 kHz/300G or 796 kHz/200 G. The magnetization values presented are normalized to the total sample mass.

In Figure 6, the SAR values of Zn_0.4_Fe_2.6_O_4_@MnFe_2_O_4_ NPs are presented as a function of their size (10, 15, 22, and 50 nm; Figure 6a). Additionally, the heating curves of 50 nm Zn_0.4_Fe_2.6_O_4_@MnFe_2_O_4_ NPs are shown as a function of time under alternating magnetic fields of 536.5 kHz/300 G and 796 kHz/200 G (Figure 6b).

The magnetic hyperthermia performance of the 50 nm Zn_0.4_Fe_2.6_O_4_@MnFe_2_O_4_ core–shell nanoparticles is demonstrated by their SAR values of 96.92 and 110.38 W·g^−1^ under alternating magnetic fields of 536 and 796 kHz, respectively (Figure 6a). These values are significant in the context of superparamagnetic, biocompatible ferrite nanoparticles and are higher than those reported in previous studies involving similar core–shell systems [16]. The corresponding heating curves (Figure 6b) show temperature increases of 6.5 and 9.6 °C after 60 s, confirming the efficiency of these nanoparticles for hyperthermia applications.

The improved magnetic hyperthermia performance observed in the Zn_0.4_Fe_2.6_O_4_@MnFe_2_O_4_ core–shell nanoparticles can be attributed to a combination of factors, including the increased particle size, enhanced magnetic moment, and the presence of a moderately anisotropic MnFe_2_O_4_ shell. It is important to note, however, that magnetic anisotropy alone does not guarantee enhanced heating efficiency. As highlighted by Dirba et al., there exists an optimal range of magnetic anisotropy for effective energy dissipation in alternating magnetic fields [22]. Excessively high anisotropy may hinder relaxation processes and reduce SAR values. In our system, the MnFe_2_O_4_ shell likely provides anisotropy within an effective range, enabling efficient Néel and Brownian relaxation while maintaining superparamagnetic behavior [23]. Thus, the observed SAR values result from a balanced interplay between particle size, saturation magnetization, magnetic anisotropy, and field parameters, rather than from any single dominant factor.

For comparison, Shabalkin et al. reported SAR values of only ≈8 W·g^−1^ for ZnFe_2_O_4_@MnFe_2_O_4_ particles with thin shells (0.5–1.7 nm) under a low magnetic field (100 Oe at 75 kHz), which are significantly lower than those obtained in our study [24]. The higher SAR values observed here can be attributed to both the optimized size and the application of higher frequencies, closer to biomedical operation ranges. Nevertheless, we acknowledge that SAR performance also depends on other factors such as crystallinity, anisotropy, and field amplitude, which are under ongoing investigation [25].

The role of size in hyperthermia performance has been further highlighted in other studies [16]. It is well established that larger core–shell NPs exhibit enhanced magnetic properties due to increased magnetic anisotropy and moment, both of which contribute to higher energy dissipation. For example, CoFe_2_O_4_@MnFe_2_O_4_ NPs have been shown to achieve SAR values in the range of 210–320 W·g^−1^ under specific magnetic field conditions, demonstrating the effectiveness of core–shell structures in enhancing hyperthermia performance [7]. While the SAR values observed for the Zn_0.4_Fe_2.6_O_4_@MnFe_2_O_4_ NPs in this study are lower than those of CoFe_2_O_4_@MnFe_2_O_4_ NPs, it is important to note that Co-based ferrites typically exhibit higher coercivity and saturation magnetization, which inherently favor higher SAR. However, despite their high SAR values, cobalt-containing nanoparticles are not suitable for biomedical applications due to the well-known cytotoxicity of cobalt ions. Studies have shown that cobalt ions released from nanoparticles can induce oxidative stress, inflammation, and cellular damage, which limits their use in clinical applications. In contrast, Zn and Mn ferrites are known for their lower cytotoxicity and biocompatibility, making them more suitable for biomedical applications. This biocompatibility advantage further emphasizes the potential of Zn_0.4_Fe_2.6_O_4_@MnFe_2_O_4_ NPs for use in hyperthermia cancer therapies.

Another key factor influencing hyperthermia performance is the core–shell structure itself. The core–shell architecture allows for the synergistic combination of the distinct magnetic properties of the core (Zn_0.4_Fe_2.6_O_4_) and the shell (MnFe_2_O_4_), leading to a stronger effective anisotropy and magnetic moment than those observed for single-phase materials [7]. This is consistent with findings from previous reports that show that bimagnetic core–shell NPs exhibit higher SAR values than single-component systems [7]. This enhancement is attributed to the interface exchange coupling effect, which improves magnetic anisotropy and, consequently, the Néel relaxation. In addition, the larger overall size of the NPs favors an increase in Brownian relaxation, which becomes more prominent at higher frequencies.

The heating efficiency of the NPs, as depicted in Figure 6b, highlights their rapid and sustained heating capability, a critical requirement for hyperthermia applications. The temperature increases of 6.5 and 9.6 °C under 536 and 796 kHz, respectively, surpass the thermal threshold required to induce apoptosis in cancer cells (42–46 °C). The fast heating kinetics observed here are comparable to those reported by Shabalkin et al., where ZnFe_2_O_4_@MnFe_2_O_4_ NPs reached the desired hyperthermic range (42 °C) within seconds under similar field conditions [24]. In contrast, other studies with single-phase MnFe_2_O_4_ or ZnFe_2_O_4_ NPs often require higher magnetic field intensities to achieve comparable heating rates, further confirming the enhanced hyperthermic properties of the core–shell configuration.

## Conclusion

In this work, we successfully synthesized a series of monodisperse Zn_0.4_Fe_2.6_O_4_ and Zn_0.4_Fe_2.6_O_4_@MnFe_2_O_4_ core–shell nanoparticles with tunable sizes using a thermal decomposition method. Structural and magnetic characterization confirmed that both particle size and core–shell architecture play critical roles in modulating magnetic properties. Notably, the saturation magnetization increased significantly with particle diameter, with the 50 nm core–shell NPs reaching ≈7.0 A·m^2^·kg^−1^, compared to 0.012 A·m^2^·kg^−1^ for their 5 nm counterparts. The addition of a MnFe_2_O_4_ shell further enhanced the magnetic response across all sizes, confirming its functional contribution to the overall magnetic behavior. Zero-field-cooled/field-cooled (ZFC/FC) analyses demonstrated the superparamagnetic nature of the particles at room temperature, with a blocking temperature of approximately 235 K for the largest core–shell system. In magnetic hyperthermia experiments, the 50 nm Zn_0.4_Fe_2.6_O_4_@MnFe_2_O_4_ NPs achieved SAR values up to 110 W·g^−1^, indicating efficient energy dissipation under alternating magnetic fields. These results highlight the importance of precise control over size and composition in designing optimized magnetic nanomaterials. While further improvements (e.g., surface modification and TGA normalization) remain to be explored, this study provides solid experimental evidence supporting the relevance of core–shell spinel ferrites for magnetic hyperthermia and related biomedical applications.

## Supporting Information

File 1 Additional figures and table.

## Data Availability

Data generated and analyzed during this study is available from the corresponding author upon reasonable request.
