# Supplementary material for: Improving magnetic properties of Mn- and Zn-doped core–shell iron oxide nanoparticles by tuning their size
Source: Beilstein J Nanotechnol. 2025 Dec 15;16:2285–95. doi: 10.3762/bjnano.16.157 (PMC12719879; doi:10.3762/bjnano.16.157)
Supplement: File 1 — Additional figures and table. [file Beilstein_J_Nanotechnol-16-2285-s001.pdf]

BEILSTEIN JOURNAL OF NANOTECHNOLOGY

Supporting Information

for

Improving magnetic properties of Mn- and Zn-doped core–shell iron oxide nanoparticles by tuning their size

Dounia Louaguef, Ghouti Medjahdi, Sébastien Diliberto, Klaus M. Seemann, Thomas Gries, Joelle Bizeau, Damien Mertz, Eric Gaffet and Halima Alem

Beilstein J. Nanotechnol. **2025,** *16,* 2285–2295. doi:10.3762/bjnano.16.157

Additional figures and table

License and Terms: This is a supporting information file under the terms of the Creative Commons Attribution License (https://creativecommons.org/licenses/by/4.0). Please note that the reuse, redistribution and reproduction in particular requires that the author(s) and source are credited and that individual graphics may be subject to special legal provisions.
The license is subject to the *Beilstein Journal of Nanotechnology* terms and conditions: (https://www.beilstein-journals.org/bjnano/terms)

a- X-ray diffraction analysis

XRD investigations reveals the structural characterization of the NPs. X-ray patterns of $Zn_{0.4}Fe_{2.6}O_4$ NPs before and after annealing at 300 °C and 700 °C are shown in Figure S1. The annealing of the nanoparticles at these temperatures was an essential step to eliminate the excess of oleic acid to distinguish the Bragg peaks.

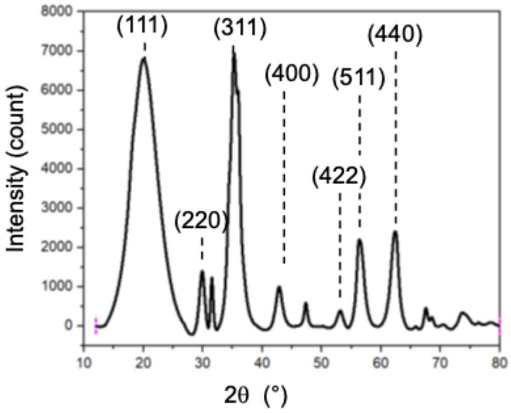

Figure S1: XRD patterns of the as-prepared $Zn_{0.4}Fe_{2.6}O_4$ NPs at 300 °C (Cu Kα wavelength).

The X-ray diffractograms of the NPs annealed at 300 °C (b) show the iron oxide phases of the NPs. They can represent either magnetite (Fe_2O_3) or maghemite (γ-Fe_2O_3), because of the similarity of their XRD patterns, it is impossible to make the difference between the $Fe_{3-\delta}O_4$ magnetite phase from the maghemite phase with the X-ray analysis [1,2]. To precise the right phase, the NPs were analyzed by Mössbauer spectroscopy in the next step, we have also compared to the as prepared nanoparticles (Figure S2).

b- Mössbauer spectroscopy

Mössbauer spectroscopy analysis were performed to determine the NPs phase. The obtained MS spectra of the as-prepared NPs and those annealed at 300 and 700 °C are shown in (Figure 2). Their hyperfine parameters are listed in Table 2.

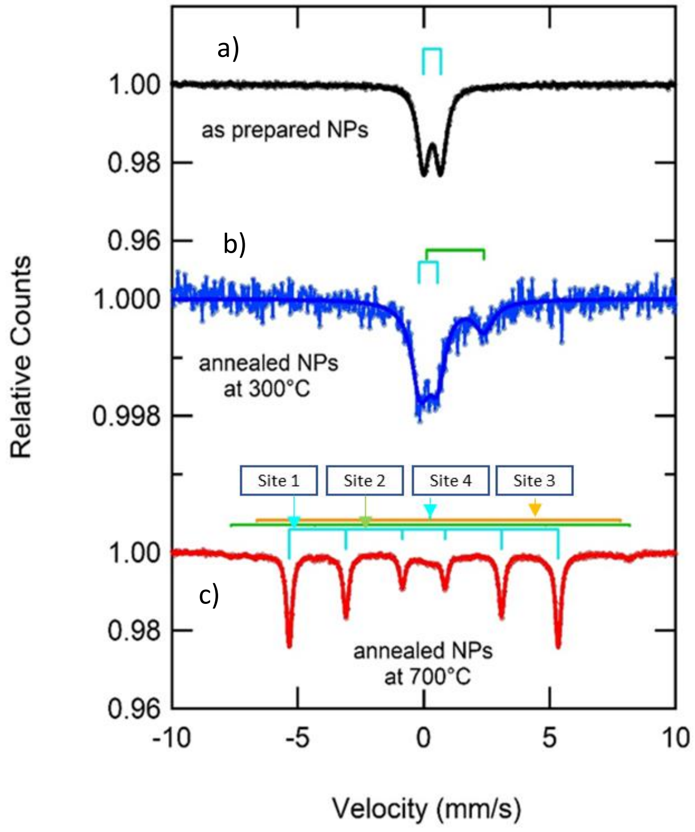

Figure S2: Mössbauer spectra of the as-prepared $Zn_{0.4}Fe_{2.6}O_4$ NPs (a), annealed NPs at 300 °C (b), and at 700 °C (c).

The as-prepared NPs Mössbauer (spectrum (a), Figure S2) represents one pure doublet which corresponds to the superparamagnetic Fe_3O_4 NPs (Iron in oxidation state III). The presence of Fe^{3+} is also shown in spectrum (spectrum(b), Figure S2) which corresponds to NPs annealed at 300 °C with a percentage of 65% and the remaining 35% represent Fe^{2+}. On the other hand, the well-defined sextet obtained from the annealed NPs at 700 °C (spectrum (c), Figure S2) (line width 0.50 mm/s) is attributed to Fe metal. The site 1 of this spectrum corresponds to Fe^0 and the percentage of this phase is 85%, the sites 2 and 3 correspond to magnetite with a percentage of 8%, finally the site 4 is attributed to 7% of superparamagnetic NPs (see Table S1).

Table S1: The hyperfine parameters of the as-prepared NPs and those annealed at 300 and 700 °C obtained from Mössbauer analysis.

T (°C)	Component (sites)	IS (mm/s)	EQ (mm/s)	*B* (T)	Phase	Mass (%)
As Prepared NPs	1	0.33	0.69	–	Fe III	–
300 °C	1	0.19	0.74	–	Fe III	65
	2	1.24	2.28	–	Fe II	35
700 °C	1	0	0	33.1	Fe^0	85
	2	0.29	0	49	$Fe_{3-\delta}O_4$	8
	3	0.62	0	46		
	4	0.25	0	_	Fe III	7

c- ZF-ZFC curves

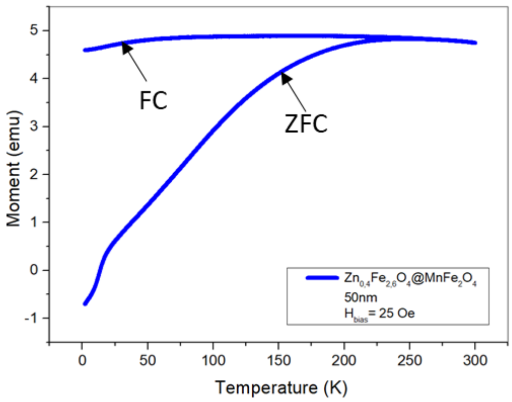

Figure S3: Zero-field-cooled (ZFC) and field-cooled (FC) magnetization curves of $Zn_{0.4}Fe_{2.6}O_4$@$MnFe_2O_4$ nanoparticles (50 nm) with surface oleic acid ligands, measured under an applied magnetic field of 25 Oe. The clear bifurcation between the ZFC and FC curves confirms the superparamagnetic behavior of the nanoparticles, with a blocking temperature observed around 235 K.

References

1. Glasgow, W.; Fellows, B.; Qi, B.; Darroudi, T.; Kitchens, C.; Ye, L.; Crawford, T. M.; Thompson Mefford, O. *Particuology* **2016**, *26*, 47–53. doi:10.1016/j.partic.2015.09.011

2. Köçkar, H.; Karaagac, O.; Özel, F. *J. Magn. Magn. Mater.* **2019**, *474*, 332–336. doi:10.1016/j.jmmm.2018.11.053
